# The role of veno-venous bypass in liver transplant

**DOI:** 10.1097/ACO.0000000000001504

**Published:** 2025-05-20

**Authors:** Elena Ahrens, Giorgia Caputo, Raymond Planinsic, Christian Zanza, Yaroslava Longhitano

**Affiliations:** a Department of Anesthesia, Critical Care and Pain Medicine, Beth Israel Deaconess Medical Center, Harvard Medical School; b Center for Anesthesia Research Excellence (CARE), Beth Israel Deaconess Medical Center, Boston, Massachusetts, USA; c Department of Anesthesia and Intensive Care, San Luigi Gonzaga Hospital, Turin, Orbassano, Italy; d Department of Anesthesiology and Perioperative Medicine, University of Pittsburgh Medical Center, Pittsburgh, Pennsylvania, USA; e Department of Systems Medicine, Geriatric Medicine Residency Program, University of Rome "Tor Vergata", Rome, Italy

**Keywords:** liver transplantation, piggyback, veno-venous bypass

## Abstract

**Purpose of review:**

Veno-venous bypass (VVB) ensures end-organ perfusion and minimizes splanchnic venous congestion during liver transplant procedures. The adoption of the piggyback technique, where flow through the inferior vena cava is preserved, has prompted a decline in the routine use of VVB. Meanwhile, recommendations on VVB use in liver transplantation remain equivocal. This article explores the clinical implications of VVB use in liver transplantation and offers a comprehensive analysis of its benefits and risks in the context of recent surgical advancements.

**Recent findings:**

Evidence indicates that patients undergoing complex procedures or with baseline renal dysfunction may benefit from VVB for conventional liver resection, emphasizing the need for careful patient selection. By contrast, small, retrospective studies suggest lower transfusion requirements and improved graft survival when the piggyback approach was used without VVB, but evidence remains sparse. While direct bypass cannulation-associated complications remain a concern, technical advancements have made VVB use increasingly safe.

**Summary:**

In conclusion, VVB remains an important tool in selected, high-acuity patients, but offers limited benefit in more stable patients undergoing piggyback liver resection. Large-scale randomized studies are needed to elucidate the benefit of VVB in selected patient populations undergoing procedures with different surgical approaches.

**KEY POINTS**

- Veno-venous bypass (VVB) remains a frequently employed tool to support liver transplantations for high-acuity patients undergoing complex procedures.
- Although sparse, evidence on VVB in piggyback procedures indicates increased blood loss and transfusion requirements without patient or graft survival benefits, highlighting the need for selective use.
- High-quality, large-scale randomized studies are warranted to clarify the risk-benefit profile of VVB across different patient populations and surgical approaches to liver transplantation.

## INTRODUCTION

**FIGURE 1.**
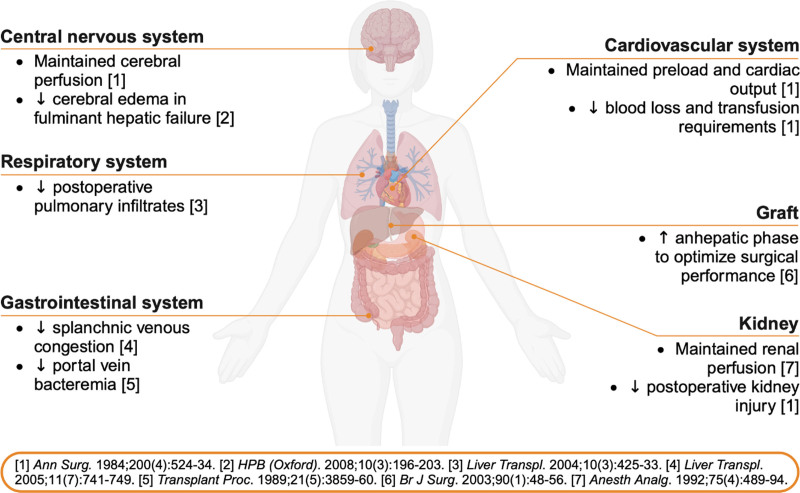
Hypothesized benefits of VVB for liver transplant. VVB, veno-venous bypass.

An estimated 10 000 patients underwent liver transplant procedures in the USA in 2022, with an over 50% increase in case volume over the last decade [1]. The conventional approach to liver transplantation, pioneered by Starzl in 1963, involved crossclamping of the inferior vena cava [2]. However, splanchnic congestion and a drastic reduction in preload and cardiac output during this intervention prompted efforts to bypass the venous circulation [3]. First introduced by Shaw *et al*. [4] in Pittsburgh in the 1980s, veno-venous bypass (VVB) quickly became a standard technique to ensure the venous return to the heart and eliminate intestinal congestion during crossclamping of the inferior vena cava in orthotopic liver transplant recipients [5,6]. Hypothesized benefits of VVB for liver transplant procedures are summarized in Fig. 1. While initial studies demonstrated up to 40% decreased blood loss, maintained hemodynamic stability, and improved postoperative outcomes including lower risk of kidney injury [4,7] (Fig. 1), the routine use of VVB declined over the past years [8]. Emerging evidence raised questions about the perioperative implications of VVB use and suggested increased transfusion requirements, risk of hypothermia, and morbidity associated with cannula placement [9–11]. Advancements in surgical approaches, such as the introduction of the piggyback technique in the 1990s, which includes preservation of the inferior vena cava [12], and additional temporary portocaval shunting [13], further reduced the need for VVB in these procedures [14]. However, little data are available on the risks and benefits of VVB after the establishment of piggyback liver transplant procedures in routine clinical care [15].

Use of VVB in orthotopic liver transplants remains subject to a large variability across centers in the USA, and recent recommendations emphasize the lack of high-quality evidence to guide its application [16,17]. However, survey data indicate that approximately 42% of transplant centers across the country reported using VVB, demonstrating its persistent relevance to the field [18]. This review article aims to summarize the current literature on the risks, benefits, and limitations of VVB for orthotopic liver transplantations in the context of recent developments in surgical techniques. We discuss strategies to improve patient outcomes by highlighting potential indications for VVB use and inform future studies about current challenges in generating high-quality evidence on this topic.

## PERIOPERATIVE OUTCOMES

### Renal function

Initial studies confirmed the hypothesized beneficial effect of VVB on renal perfusion during the anhepatic phase of liver transplantation. Veroli and colleagues demonstrated a decrease in renal perfusion pressure and urine output (1 ml/kg/h decrease from dissection to anhepatic phase) in patients not receiving VVB, which could be prevented when a bypass was established [19]. These findings were corroborated by Grande *et al*. [20], who reported a reduction in inulin clearance, a surrogate of glomerular filtration rate, in 77 patients randomized to undergo liver transplantation without, compared to with VVB. However, both aforementioned studies demonstrated no difference in short-term postoperative kidney function based on hemodialysis requirement or creatinine levels, raising questions about the clinical relevance of these findings [19,20]. Multiple studies, including a 5-year retrospective study targeting 424 liver transplant recipients [21], confirmed these observations by demonstrating comparable incidences of postoperative renal failure in patients with normal preoperative kidney function undergoing conventional liver transplantation with and without VVB [22–24].

Of note, one recent study in nonpiggyback liver transplantation showed that in patients with compromised pretransplant renal function (baseline creatinine ≥1.2 mg/dl), VVB significantly reduced the incidence of postoperative acute kidney injury (37.2% vs. 50.8%), which was not observed in patients with normal baseline kidney function [25]. In contrast to previous studies, Sun and colleagues controlled for a variety of potential confounding variables, setting their study apart from earlier reports [22–24]. These data suggest that patients undergoing conventional liver transplantation with baseline impairment in kidney function may benefit from the use of VVB.

Trends of favoring the piggyback approach over conventional retrocaval hepatic resection prompted research on the interplay between the surgical approach and VVB use. For example, Cabezuelo *et al*. [26] demonstrated the standard surgical technique with or without VVB, compared to the piggyback technique, to be an independent risk factor for postoperative acute renal failure defined as serum creatinine >1.5 mg/dl, a creatinine increase by 50% or more from baseline, or oliguria requiring renal replacement therapy, indicating that the piggyback approach should be implemented where possible.

Yet, evidence on the association between VVB use and postoperative kidney function in piggyback procedures remains equivocal, and nonstandardized definitions of postoperative kidney failure make it difficult to directly compare studies (Table 1). A small study reported no difference in post-transplant kidney injury in unadjusted analyses on 900 patients with over 90% adherence to the piggyback technique [29^■■^]. By contrast, a 2024 propensity score weighted analysis in piggyback-only cases demonstrated higher risks for acute kidney injury during the first 24 h after ICU admission together with 85% higher odds for renal replacement therapy needed during ICU stay [30^■■^], suggesting that VVB may not have beneficial effects in these procedures.

**Table 1.** Retrospective data on implications of veno-venous bypass for perioperative transfusion requirements and renal function

Results with VVB	Definition of renal outcome	Study design	Reference
Retrocaval hepatic resection			
= Postop. kidney failure ↑ Intraop. intravenous fluid, homologous blood, FFPs, and platelets	Oliguria and requirement of hemodialysis —	Cohort study in 72 patients (VVB+ vs. VVB−). Univariate analyses	Fan *et al*. [22]
= Postop. acute kidney injury	≥50% ↑ in creatinine from baseline or absolute ↑ ≥0.3 mg/dl within 48 h	Cohort study in 424 patients (VVB+ vs. VVB−). Univariate analyses	Hilmi *et al*. [21]
↓ Postop. acute kidney injury, if baseline dysfunction present	≥50% ↑ in creatinine from baseline or absolute ↑ ≥0.3 mg/dl within 48 h	Cohort study in 1037 patients (VVB+ vs. VVB−). Propensity-score matching and multivariable analyses	Sun *et al*. [25]
= Postop. acute kidney injury = Intraop. number of PRBCs and FFPs	≥50% ↑ in creatinine from baseline or absolute ↑ ≥0.3 mg/dl within 48 h —	Cohort study in 38 patients (VVB+ vs. VVB−). Univariate analyses	Guarino *et al*. [23]
Piggyback (PGB) or retrocaval hepatic resection			
↑ Postop. acute kidney failure (irrespective of PGB) ↑ Intraop. number of PRBCs and FFPs (irrespective of PGB)	≥200% ↑ in creatinine from baseline within 48 h	Cohort study in 426 patients (VVB+ in PGB− vs. VVB+ or VVB− in PGB+). Univariate analyses	Sakai *et al*. [27]
↑ Postop. acute kidney failure with PGB− irrespective of VVB but ↑ postop. creatinine decreases in PGB–VVB+ ↑ Intraop. number of PRBCs and FFPs with PGB− (irrespective of VVB)	absolute ↑ in creatinine ≥0.5 mg/dl on day 1–14	Cohort study in 414 patients (VVB+ or VVB− in PGB− vs. VVB− in PGB+). Univariate analyses.	Schmitz *et al*. [28]
↑ Intraop. number of PRBCs and FFPs (irrespective of PGB)	—	Cohort study in 242 patients (VVB+ vs. VVB− in PGB− and PGB+). Univariate analyses	Kim *et al*. [8]
Piggyback (PGB) hepatic resection			
= Postop. acute kidney injury ↑ Intraop. number of PRBCs and FFPs	≥50% ↑ in creatinine from baseline or absolute ↑ ≥0.3 mg/dl within 48 h —	Cohort study in 900 patients (VVB+ vs. VVB−). Univariate analyses	Weinberg *et al*. [29^■■^]
↑ Postop. acute kidney injury ↑ Intraop. massive PRBC transfusion	Requirement of hemodialysis during ICU stay, or ≥100% ↑ in creatinine from baseline within 48 h	Cohort study in 874 patients (VVB+ vs. VVB−). Propensity-score matching and multivariable analyses	Laici *et al*. [30^■■^]

FFP, fresh frozen plasma; PGB, piggyback; PRBC, packed red blood cells; VVB, veno-venous bypass.

### Transfusion requirements and blood loss

A presumed reduction in blood loss and resuscitation requirements was part of the initial rationale for the widespread adoption of VVB during orthotopic liver transplantations (Fig. 1). A small meta-analysis including three randomized controlled trials conducted in the 1990s reported no difference in blood transfusion requirements between VVB and control groups [17]. However, more recent data from a prospective cohort study in 72 liver transplant recipients demonstrated a 1600 ml higher amount of intravenous fluid resuscitation volume, along with almost 50% increased requirements for packed red blood cell, platelet, and fresh frozen plasma units among patients undergoing VVB [22]. These findings align with reports from an Australian study in 900 patients, where the number of units of packed red blood cells transfused was 7.0 (4.8–12.5) units in the VVB group compared to 3.0 units (1.0–6.0) in the non-VVB group [29^■■^]. Notably, a study in 27 patients undergoing liver resection under prolonged (≥55 min) vascular exclusion with hypothermic liver perfusion demonstrated a more than 60% lower blood loss (600 vs. 1,750 ml) under VVB [31]. While these data do not directly pertain to liver transplantation, they emphasize that the technique may still be considered in select high-acuity patients where the anatomical situation requires complete clamping of the inferior vena cava.

Sakai and coworkers reported a reduction in perioperative red blood cell (*P* = 0.006), fresh frozen plasma (*P* = 0.005), and cell saver return (*P* = 0.007) requirements in 426 patients undergoing piggyback liver resection without VVB [27]. These beneficial effects of the piggyback approach were not observed when a VVB was used, suggesting that the potential benefits of the piggyback technique for transfusion requirements were partially attributable to the elimination of VVB. Another retrospective study spanning over a 3-year VVB-weaning period corroborates these findings by reporting a decrease in blood product requirements with increasing implementation of non-VVB piggyback liver resection [8]. However, the study did not account for other factors resulting from potential changes in practice that could have impacted the observed trend in transfusion requirements. While these unadjusted data provide a weak level of evidence (Table 1), they suggest that the use of VVB may be unnecessary in procedures where the piggyback approach can be used.

### Graft outcomes

Establishment of a VVB requires substantial expertise and has been shown to be associated with a prolonged total operative duration of up to 3 h [22], which can in turn affect short- and long-term graft function [32]. In a retrospective analysis on 414 patients, Schmitz and colleagues found no difference in graft function within 2 weeks, quantified via bilirubin and transaminase levels, between patients undergoing complete clamping of the inferior vena cava with or without VVB [28]. However, despite an often described shorter operative and ischemic time in patients undergoing piggyback liver transplant procedures without VVB [33], this technique was also not associated with improved graft survival in univariate analysis. Only one study reported a lower risk of 30-day graft loss (3.4%) with the piggyback technique without VVB, compared to procedures employing VVB irrespective of surgical approach (12.5 and 11.5% for retrohepatic caval resection and piggyback with VVB, respectively) [27]. However, this study was a retrospective analysis from a single institution, where the choice of bypass use and surgical approach was to the discretion of the attending surgeon. Thus, these data give a hint on potential benefits of the piggyback technique that results from the avoidance of VVB, but cannot be generalized to other settings and patients.

For example, multiple studies with thorough adjustment for underlying comorbidity load of patients undergoing exclusively piggyback liver transplant procedures reported no benefit of VVB avoidance for graft function [29^■■^,30^■■^]. These findings indicate that while VVB may benefit high-risk patients in conventional procedures, its utility in improving graft-related outcomes in the piggyback technique remains less clear, advocating for patient-centered approaches in perioperative planning.

### Perioperative mortality

Multiple early randomized studies investigated patient survival after orthotopic liver transplantation with vs. without VVB. Grande and colleagues reported no difference in early postoperative mortality within 30 days between patients with, vs. without VVB in their 1996 randomized controlled trial [20]. A recent retrospective study in 1037 patients undergoing nonpiggyback orthotopic liver transplant procedures with and without VVB supported these findings by demonstrating comparable 1-year recipient survival among patients with impaired preoperative kidney function following multivariable regression analyses [25].

By contrast, one small study demonstrated a slightly higher incidence of in-hospital mortality when a VVB was established, which marginally reached statistical significance in nonparametric tests (*P* = 0.042) [22]. However, the 72 study patients were not randomly, but sequentially allocated to the treatment groups, resulting in notable imbalances (e.g. 31.9% higher absolute rate of preoperative ICU admission of VVB patients), which may have additionally limited the finding’s generalizability [22]. Another recent study in patients undergoing liver transplant procedures using the piggyback technique alarmed by reporting excessive rates of postoperative mortality in VVB patients [29^■■^]. Weinberg and coworkers documented a 17.4% higher absolute risk of in-hospital mortality among patients receiving an intraoperative VVB [29^■■^]. Of note, the VVB was established as a rescue intervention for massive intraoperative bleeding in 40.1% of affected patients, emphasizing the relevance of addressing unobserved confounding in studies evaluating the risks and benefits of this technique. While the authors of these two studies provide important information on the availability and applicability of VVB as a rescue tool in complex cases of orthotopic liver transplant procedures [34], their unadjusted analyses in imbalanced groups preclude meaningful conclusions on VVB-associated mortality risk in the more general population. By contrast, Laici and colleagues applied propensity score-weighted general linear models accounting for patient- and procedure-related characteristics that may affect the decision whether or not to establish a VVB [30^■■^]. These well-analyzed data showed no association between VVB use and in-hospital mortality [30^■■^]. While Sakai and coworkers supported these findings of comparable mortality even irrespective of surgical approach [27], the disparity in findings of unadjusted vs. adjusted analyses and lack of studies investigating long-term outcomes underscores the complexity of interpreting available evidence.

### Direct bypass and cannulation-associated complications

The conventional approach to cannula placement in the axillary vein involved a surgical cut-down, which itself is associated with a considerable surgical risk. First introduced by Oken in 1994 [35], percutaneous insertion of an 18 Fr venous return cannula, performed under ultrasound guidance via the Seldinger technique, has become a de facto standard of care across institutions in the USA [36,37]: A 1999 randomized controlled trial on 40 liver transplant procedures demonstrated a 59 min longer operative duration in patients receiving surgical, compared to percutaneous cannula placement [38]. Another study in 81 patients undergoing liver transplantations reported a lower risk of axillary and groin lymphoceles while similar intraoperative blood flow rates were achieved (2238 vs. 2197 ml/min) [39], suggesting that percutaneous cannulation may not only be faster to achieve, but also safer. Still, multiple case reports describe bypass and cannulation-associated mortality, for example, due to hemothorax development on VVB initiation [40], and life-threatening morbidity [41] including the occurrence of air embolisms [42]. These risks underscore the importance of careful patient selection and vigilance during and after cannulation. Nevertheless, safety standards in the operating room have increased over the last decades and are still increasing [43], and prospective evidence suggest that complications associated with bypass placement and percutaneous cannulation remain rare [44].

## OUTLOOK

Despite an overall decrease in its application in recent years, especially in the context of the transition to the piggyback technique [8,45], VVB remains a widely used tool in liver transplant procedures [18]. Early evidence from randomized studies demonstrated the beneficial effects of VVB in procedures with total clamping of the vena cava inferior, while current recommendations regarding its utility in piggyback liver transplant procedures are mostly based on small, retrospective studies. These report longer operative times, higher transfusion requirements, a risk of cannulation-associated complications and no benefit for patient or graft survival, but are often limited by the lack of rigorous methods of confounder control to account for imbalance in baseline characteristics. This is particularly relevant in the context of the increasing popularity of piggyback procedures where VVB is used as a rescue intervention [34], and recent evidence suggesting that VVB may be most beneficial for high-acuity patients undergoing complex procedures. Large-scale randomized studies comparing the outcomes of VVB in patients with different surgical approaches and depending on baseline patient characteristics are needed to guide its future use in orthotopic liver transplantation.

## CONCLUSION

In the context of recent surgical advances including the widely established surgical preservation of the inferior vena cava and percutaneous cannulation, VVB remains a frequently employed technique in more complex cases of liver transplantation. Recent high-quality evidence on the benefits of VVB in the more general population undergoing liver transplant procedures is lacking. Data suggest a varying risk-benefit ratio depending on baseline patient characteristics and surgical approach, urging clinicians to selectively use VVB. High-acuity patients, especially when the piggyback technique cannot be employed, may benefit most from VVB use. We strongly encourage future large-scale randomized trials comparing outcomes of VVB use in selected patient populations with a varying comorbidity burden undergoing procedures with different surgical approaches.

## Acknowledgements

*We extend our deepest gratitude to the esteemed Italian-Americans, Leonardo Domiziano Zanza and Lucrezia Flavia Zanza, for their invaluable assistance in revising and editing our manuscript.*

## Financial support and sponsorship

*None.*

## Conflicts of interest

*There are no conflicts of interest.*

## References

[1] Kwong AJ Kim WR Lake JR. OPTN/SRTR 2022 annual data report: liver. Am J Transplant 2024; 24(2S1):S176–S265. 38431359 10.1016/j.ajt.2024.01.014

[2] Starzl TE Marchioro TL Vonkaulla KN. Homotransplantation of the liver in humans. Surg Gynecol Obstet 1963; 117:659–676. 14100514 PMC2634660

[3] Calne RY Smith DP McMaster P. Use of partial cardiopulmonary bypass during the anhepatic phase of orthotopic liver grafting. Lancet 1979; 2:612–614. 90273 10.1016/s0140-6736(79)91668-4

[4] Shaw BW Martin DJ Marquez JM. Venous bypass in clinical liver transplantation. Ann Surg 1984; 200:524–534. 6385876 10.1097/00000658-198410000-00013 PMC1250523

[5] Kaufman RD Khoury GF. Hemodynamic changes with initiation of veno-venous bypass in orthotopic liver transplant patients. Am J Anesthesiol 1995; 22:184–188. 10150762

[6] Neuhaus P Platz KP. Liver transplantation: newer surgical approaches. Baillieres Clin Gastroenterol 1994; 8:481–493. 8000095 10.1016/0950-3528(94)90033-7

[7] Shaw BW Martin DJ Marquez JM. Advantages of venous bypass during orthotopic transplantation of the liver. Semin Liver Dis 1985; 5:344–348. 3909428 10.1055/s-2008-1040631 PMC3008817

[8] Kim HY Ko JS Joh J. Weaning of veno-venous bypass in liver transplantation: a single center experience. Transplant Proc 2018; 50:2657–2660. 30401371 10.1016/j.transproceed.2018.03.075

[9] Neelakanta G Colquhoun S Csete M. Efficacy and safety of heat exchanger added to venovenous bypass circuit during orthotopic liver transplantation. Liver Transpl Surg 1998; 4:506–509. 9791162 10.1002/lt.500040610

[10] Sakai T Planinsic RM Hilmi IA Marsh JW. Complications associated with percutaneous placement of venous return cannula for venovenous bypass in adult orthotopic liver transplantation. Liver Transpl 2007; 13:961–965. 17600351 10.1002/lt.21072

[11] Hilmi IA Planinsic RM. Con: venovenous bypass should not be used in orthotopic liver transplantation. J Cardiothorac Vasc Anesth 2006; 20:744–747. 17023301 10.1053/j.jvca.2006.06.004

[12] Tzakis A Todo S Starzl TE. Orthotopic liver transplantation with preservation of the inferior vena cava. Ann Surg 1989; 210:649–652. 2818033 10.1097/00000658-198911000-00013 PMC1357802

[13] Belghiti J Noun R Sauvanet A. Temporary portocaval anastomosis with preservation of caval flow during orthotopic liver transplantation. Am J Surg 1995; 169:277–279. 7840394 10.1016/S0002-9610(99)80151-2

[14] Chari RS Gan TJ Robertson KM. Venovenous bypass in adult orthotopic liver transplantation: routine or selective use? J Am Coll Surg 1998; 186:683–690. 9632158 10.1016/s1072-7515(98)00101-x

[15] Lapisatepun W Lapisatepun W Agopian V Xia VW. Venovenous bypass during liver transplantation: a new look at an old technique. Transplant Proc 2020; 52:905–909. 32113694 10.1016/j.transproceed.2020.01.048

[16] Shaker TM Eason JD Davidson BR; ERS4OLT.org Working Group. Which cava anastomotic techniques are optimal regarding immediate and short-term outcomes after liver transplantation: a systematic review of the literature and expert panel recommendations. Clin Transplant 2022; 36:e14681. 35567584 10.1111/ctr.14681 PMC10078200

[17] Gurusamy KS Koti R Pamecha V Davidson BR. Veno-venous bypass versus none for liver transplantation. Cochrane Database Syst Rev 2011:CD007712. 10.1002/14651858.CD007712.pub2 21412907

[18] Crouch C Sakai T Aniskevich S. Adult liver transplant anesthesiology practice patterns and resource utilization in the United States: survey results from the society for the advancement of transplant anesthesia. Clin Transplant 2022; 36:e14504. 34637561 10.1111/ctr.14504

[19] Veroli P el Hage C Ecoffey C. Does adult liver transplantation without venovenous bypass result in renal failure? Anesth Analg 1992; 75:489–494. 1530159 10.1213/00000539-199210000-00004

[20] Grande L Rimola A Cugat E. Effect of venovenous bypass on perioperative renal function in liver transplantation: results of a randomized, controlled trial. Hepatology 1996; 23:1418–1428. 8675159 10.1002/hep.510230618

[21] Hilmi IA Damian D Al-Khafaji A. Acute kidney injury following orthotopic liver transplantation: incidence, risk factors, and effects on patient and graft outcomes. Br J Anaesth 2015; 114:919–926. 25673576 10.1093/bja/aeu556

[22] Fan ST Yong BH Lo CM. Right lobe living donor liver transplantation with or without venovenous bypass. Br J Surg 2003; 90:48–56. 12520574 10.1002/bjs.4026

[23] Guarino G Licitra G Ghinolfi D. Use of an intraoperative veno-venous bypass during liver transplantation: an observational, single center, cohort study. Minerva Anestesiol 2022; 88:554–563. 35381833 10.23736/S0375-9393.22.15749-4

[24] Moreno-Gonzalez E Meneu-Diaz JG Fundora Y. Advantages of the piggy back technique on intraoperative transfusion, fluid compsumption, and vasoactive drugs requirements in liver transplantation: a comparative study. Transplant Proc 2003; 35:1918–1919. 12962848 10.1016/s0041-1345(03)00600-6

[25] Sun K Hong F Wang Y. Venovenous bypass is associated with a lower incidence of acute kidney injury after liver transplantation in patients with compromised pretransplant renal function. Anesth Analg 2017; 125:1463–1470. 28742776 10.1213/ANE.0000000000002311

[26] Cabezuelo JB Ramirez P Acosta F. Does the standard vs piggyback surgical technique affect the development of early acute renal failure after orthotopic liver transplantation? Transplant Proc 2003; 35:1913–1914. 12962846 10.1016/s0041-1345(03)00598-0

[27] Sakai T Matsusaki T Marsh JW. Comparison of surgical methods in liver transplantation: retrohepatic caval resection with venovenous bypass (VVB) versus piggyback (PB) with VVB versus PB without VVB. Transpl Int 2010; 23:1247–1258. 20723178 10.1111/j.1432-2277.2010.01144.x

[28] Schmitz V Schoening W Jelkmann I. Different cava reconstruction techniques in liver transplantation: piggyback versus cava resection. Hepatobiliary Pancreat Dis Int 2014; 13:242–249. 24919606 10.1016/s1499-3872(14)60250-2

[29■■] Weinberg L Caragata R Hazard R. Venovenous bypass in adult liver transplant recipients: a single-center observational case series. PLoS One 2024; 19:e0303631. 38820491 10.1371/journal.pone.0303631 PMC11142538

[30■■] Laici C Gamberini L Allegri D. The effects of venovenous bypass use in liver transplantation with piggyback technique: a propensity score-weighted analysis. Intern Emerg Med 2024; 19:1405–1414. 38334833 10.1007/s11739-024-03530-w

[31] Navez J Cauchy F Dokmak S. Complex liver resection under hepatic vascular exclusion and hypothermic perfusion with versus without veno-venous bypass: a comparative study. HPB (Oxford) 2019; 21:1131–1138. 30723061 10.1016/j.hpb.2018.12.012

[32] Lee DD Li J Wang G. Looking inward: The impact of operative time on graft survival after liver transplantation. Surgery 2017; 162:937–949. 28684160 10.1016/j.surg.2017.05.010

[33] Nishida S Nakamura N Vaidya A. Piggyback technique in adult orthotopic liver transplantation: an analysis of 1067 liver transplants at a single center. HPB (Oxford) 2006; 8:182–188. 18333273 10.1080/13651820500542135 PMC2131682

[34] Reddy KS Johnston TD Putnam LA. Piggyback technique and selective use of veno-venous bypass in adult orthotopic liver transplantation. Clin Transplant 2000; 14:370–374. 10946773 10.1034/j.1399-0012.2000.14040202.x

[35] Oken AC Frank SM Merritt WT. A new percutaneous technique for establishing venous bypass access in orthotopic liver transplantation. J Cardiothorac Vasc Anesth 1994; 8:58–60. 8167287 10.1016/1053-0770(94)90013-2

[36] Sakai T Gligor S Diulus J. Insertion and management of percutaneous veno-venous bypass cannula for liver transplantation: a reference for transplant anesthesiologists. Clin Transplant 2010; 24:585–591. 19930407 10.1111/j.1399-0012.2009.01145.x

[37] Washburn WK Lewis WD Jenkins RL. Percutaneous venovenous bypass in orthotopic liver transplantation. Liver Transpl Surg 1995; 1:377–382. 9346616 10.1002/lt.500010608

[38] Tisone G Mercadante E Dauri M. Surgical versus percutaneous technique for veno-venous bypass during orthotopic liver transplantation: a prospective randomized study. Transplant Proc 1999; 31:3162–3163. 10616425 10.1016/s0041-1345(99)00770-8

[39] Johnson SR Marterre WF Alonso MH Hanto DW. A percutaneous technique for venovenous bypass in orthotopic cadaver liver transplantation and comparison with the open technique. Liver Transpl Surg 1996; 2:354–361. 9346676 10.1002/lt.500020505

[40] Jankovic Z Boon A Prasad R. Fatal haemothorax following large-bore percutaneous cannulation before liver transplantation. Br J Anaesth 2005; 95:472–476. 16085686 10.1093/bja/aei216

[41] Budd JM Isaac JL Bennett J Freeman JW. Morbidity and mortality associated with large-bore percutaneous venovenous bypass cannulation for 312 orthotopic liver transplantations. Liver Transpl 2001; 7:359–362. 11303297 10.1053/jlts.2001.22708

[42] Viana JS Furtado E Romero A Furtado AL. Air embolism as a complication of venovenous bypass during liver transplant for diffuse hemangiomatosis. Transplant Proc 2003; 35:1128–1130. 12947886 10.1016/s0041-1345(03)00336-1

[43] Gehrer F Hansen D Konrad CJ. Anaesthesia related complications - a single centre data analysis at a tertiary hospital in central Switzerland. Swiss Med Wkly 2022; 152:w30169. 35752967 10.4414/smw.2022.w30169

[44] Mossdorf A Ulmer F Junge K. Bypass during liver transplantation: anachronism or revival? Liver transplantation using a combined venovenous/portal venous bypass-experiences with 163 liver transplants in a newly established liver transplantation program. Gastroenterol Res Pract 2015; 2015:967951. 25821462 10.1155/2015/967951 PMC4363615

[45] Levi DM Pararas N Tzakis AG. Liver transplantation with preservation of the inferior vena cava: lessons learned through 2,000 cases. J Am Coll Surg 2012; 214:691–8; discussion 698. 22364695 10.1016/j.jamcollsurg.2011.12.039

